# Primary malignant melanoma of the duodenum without visible melanin pigment: a mimicker of lymphoma or carcinoma

**DOI:** 10.1186/1746-1596-7-74

**Published:** 2012-06-26

**Authors:** Hongxia Li, Qinhe Fan, Zhen Wang, Hai Xu, Xiao Li, Weiming Zhang, Zhihong Zhang

**Affiliations:** 1 Department of Pathology, the First Affiliated Hospital of Nanjing Medical University, 300 Guangzhou Road, Nanjing 210029, P. R. China; 2 Department of Radiology, the First Affiliated Hospital of Nanjing Medical University, 300 Guangzhou Road, Nanjing 210029, P. R. China

**Keywords:** Malignant melanoma, Primary, Duodenum, Misdiagnosis

## Abstract

Primary malignant melanoma of the duodenum is an unusual oncologic entity. Patients usually present the similar clinical symptoms like other common tumors in this site. And there are no specific radiological features either. The cases with only little melanin pigment or without notable melanin pigment are very misleading, especially in small biopsies or frozen sections. Definite diagnosis depends on both careful histologic examination and the use of proper immunohistochemical stains. Moreover, detailed history and thorough investigation should be made to exclude the preexistence or coexistence of a primary lesion elsewhere. Herein we report the case of a 60-year-old male patient with primary malignant melanoma of the duodenum, which was misdiagnosed as lymphoma or undifferentiated carcinoma in frozen consultation. The patient had achieved disease-free survival for more than 46 months without any evidence of recurrence after surgery.

**Virtual slides:**

The virtual slides for this article can be found here: http://www.diagnosticpathology.diagnomx.eu/vs/1221457317710561.

## Background

Malignant melanoma, originated from melanocyte, is not a common tumor and accounts for 1 to 3% of all malignancies [1]. However, it is the most common metastatic tumor of the gastrointestinal (GI) tract especially the small intestine and can present with fairly common constitutional symptoms [2]. Primary malignant melanoma originating in the small bowel, particularly in the duodenum, is extremely rare and very controversial. In addition, some cases with little melanin pigment or without visible melanin pigment are very misleading, especially in small biopsy specimens or frozen sections. Identifying areas showing possible melanin pigment and immunostains with specific markers for melanoma, such as HMB45, Melan-A and S-100 protein, are diagnostically important. We encountered a case of primary malignant melanoma of the duodenum (PMMD) without any visible melanin pigment and misdiagnosed as lymphoma or undifferentiated carcinoma in frozen consultation, to highlight its clinic-pathological features and the diagnostic pitfalls.

## Case presentation

### Clinical summary

**Figure 1:**
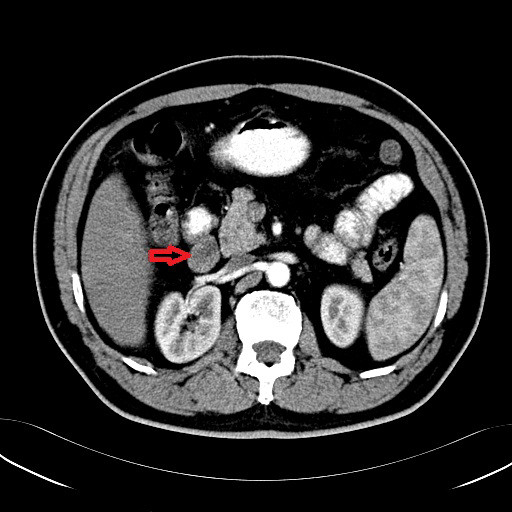
Computed tomography scan of the abdomen reveals a space-occupying lesion originating from the descending part of the duodenum (red arrow).

A 60-year-old Chinese male patient suffering from durative right abdominal pain and dark stools for one month, a recent episode of nausea and vomiting, was admitted to hospital in May 27, 2008. No history of fever, anorexia, hematemesis, radiating pain or weight loss was reported. Upper GI endoscopy revealed a malignant tumor at the descending part of the duodenum and the biopsy suspected poorly differentiated carcinoma. But there were no enough tumor cells for further immunohistochemical stain. In physical examination, there was no any obvious black nevus or nodule, nor swelled superficial lymph node. Routine hematological and biochemical studies showed no abnormalities. Serum detection showed tumor markers such as alpha fetoprotein (AFP), carcinoembryonic antigen (CEA), carbohydrate antigen 50 (CA-50), carbohydrate antigen 19–9 (CA-19-9) were all in the normal range. FOB (Fecal Occult Blood) Testing was positive. GI tract barium meal revealed a filling defect in the descendant duodenum, without obvious mucosal destruction and barium passed smoothly. Computed tomography scan of the abdomen disclosed the presence of a space-occupying lesion originating from the descending part of the duodenum with stenotic lumens (Figure 1). An exploratory laparotomy was performed through a midline incision in Jun 2, 2008 and confirmed the presence of a solid tumor arising from the lateral part of the descendant duodenum, which invading the duodenal serosa. Enlarged mesentery lymph node and peripancreatic lymph node were identified. Because the surgeons suspected the tumor as lymphoma, so only a palliative operation (tumor resection) was done.

### Pathologic findings

**Figure 2:**
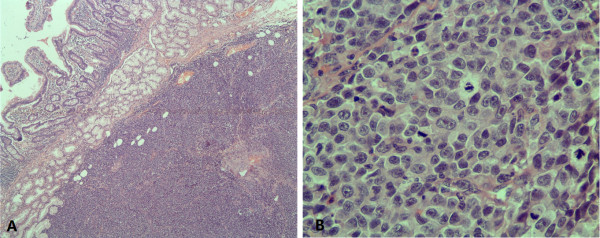
** Histological examination (hematoxylin-eosin). **(**A**) The lesion located under the enteric mucosa with diffusely infiltrative tumor cells without obvious mucosal destruction (Objective × 4); (**B**) The tumor cells showed markedly cytologic atypia with large eosinophilic nucleoli, abundant mitotic figures and moderate cytoplasm, however, without visible melanin pigment (Objective × 40).

Intraoperative consultation showed a tumor measuring 2.5 cm × 1.5 cm × 1 cm, mesentery lymph node and peripancreatic lymph node measuring 3 cm, 2 cm in diameter respectively. The cut surfaces of the specimens were both pliable and gray red without pigment. Frozen sections were applied. Histologically, the lesion located under the enteric mucosa with diffusely infiltrative tumor cells, however, without obvious mucosal destruction (Figure 2A). The tumor cells were round, oval or polygonal epithelioid, showing markedly cytologic atypia with large eosinophilic nucleoli, abundant mitotic figures and moderate cytoplasm. But there was no obviously visible melanin pigment in cytoplasm (Figure 2B). Mesentery lymph node was positive for tumor cells (1/1), whereas peripancreatic lymph node was negative (0/1). According to above-mentioned features, pathologists consulted malignant tumor, prone to lymphoma or undifferentiated carcinoma. And the surgeons thought it might be lymphoma so only local tumor was resected.

**Figure 3:**
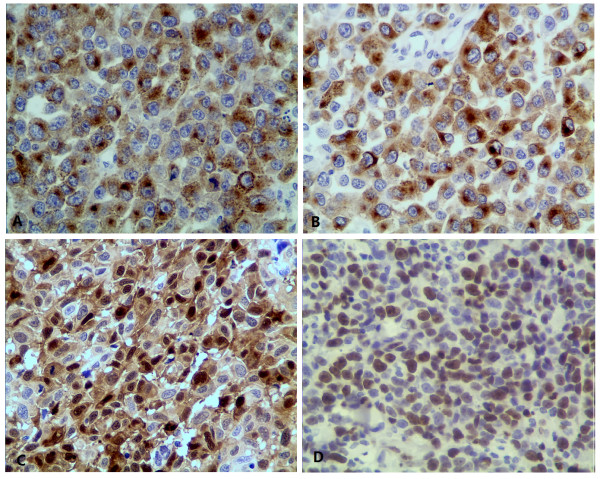
** Immunohistochemical stains (Envision, Objective × 40). **(**A**) The tumor cells were positive for HMB45 in cytoplasm; (**B**) The tumor cells were positive for Melan-A in cytoplasm; (**C**) The tumor cells were positive for protein S-100 protein in both nucleus and cytoplasm; (**D**) Ki-67 immunostaining showed a proliferative index of 60%.

Subsequently, routine hematoxylin-eosin sections (formalin-fixed and paraffin-embedded) and immunostains were done. But the results of immunohistochemical stains didn’t support the diagnoses of lymphoma and undifferentiated carcinoma. The tumor cells were strongly positive for melanoma marker (HMB45), Melan-A, S-100 protein (Figure 3A-C) and vimentin, whereas CD20, CD45RO, CD3, CD79α, myeloperoxidase, terminal deoxynucleotidyl transferase, pan cytokeratin (AE1/AE3), keratin 5/6, keratin 7, synaptophysin, chromogranin A, and sarcoma markers such as smooth muscle actin, desmin, CD117, CD34 were all negative. A proliferative index of 60% was noted with Ki-67 immunostaining (Figure 3D). So the diagnosis of malignant melanoma was confirmed.

Thorough postoperative systemic evaluations including a detailed history, clinical examination, endoscopic assessment, and radiologic imaging were done to rule out the presence of a primary cutaneous, anal, or ocular lesion, or a melanoma in any other site; however, no primary site was found. Therefore, the resected tumor was determined to represent a PMMD with mesentery lymph node metastasis. The patient refused chemotherapy and just used Chinese traditional medicine treatment intermittently to improve the immunity. Close follow-up showed that the patient is doing well with no evidence of loco regional recurrence or distant metastasis for more than 46 months after surgery.

## Discussion

Malignant melanoma is a relatively rare tumor comprising 1-3% of all tumors and exhibits an unusual tendency to metastasize to the GI tract [3]. Although frequently seen in autopsy series in up to 50 to 60% of patients, only 2 to 5% of patients are diagnosed with metastatic malignant melanoma to the GI tract while they are still alive [4]. It is because that there are no specific symptoms of early development, which are general and constitutional; and even if metastases do occur, they are usually accompany with symptoms such as abdominal pain (62%), hemorrhage (50%), nausea and vomiting (26%), mass (22%), intestinal obstruction (18%), or intussusceptions (15%), and are frequently diagnosed in an emergency situation [5]. Metastasis to the GI tract is seen most frequently in the small intestine, followed by the colon, stomach, and rectum, but is rare in the esophagus. However, primary malignant melanoma originating in the small intestine and particularly in the duodenum is extremely rare, with only a few case reports [1,6,7].

There are different theories concerning the origin of primary malignant melanoma in the small bowel, though the controversy still exists over the presence of primary malignant melanoma in this area. Some proposes that malignant melanoma may originate from neural crest cells. These multipotential cells migrate through the body and can get to the bowel via the umbilical-mesenteric canal, where they later differentiate into specialized cells [8]. Although not consistently confirmed, another theory suggests that malignant melanoma might potentially develop from amine precursor uptake and decarboxylation (APUD) cells [9]. Besides, it is presumed that the small intestine normally contains melanoblasts, which may support the assumption of primary development of melanoma at this site.

Before making the diagnosis of primary malignant melanoma of the small bowel, thorough systemic examination must be done to rule out the possibility of metastasis from other sites, which may preferentially develop on the skin, retina, anal canal, or under the nail, and less frequently at other locations such as the esophagus, penis, or vagina. In addition, there should be no history of previous removal or spontaneous regression of any atypical melanocytic skin tumor [10]. The following diagnostic criteria have been occasionally proposed. According to Sachs *et al.*[8], primary malignant melanoma in the small bowel is diagnosed where there are: 1) biopsy-proven melanoma from the intestine at a single focus; 2) no evidence of disease in any other organs including the skin, eye and lymph nodes outside the region of drainage at the time of diagnosis; and 3) disease-free survival of at least 12 months after diagnosis. According to Blecker [2], it is diagnosed when there is lack of concurrent or previous removal of a melanoma or melanocytic lesion from the skin and lack of any other organ involvement and in situ change in the overlying or adjacent GI epithelium. In this case, one single focus located in the lateral of the descendant duodenum, and thorough postoperative investigation including a detailed history, clinical examination, endoscopic assessment, and radiologic imaging failed to reveal any evidence of a pre-existing primary tumor or any other metastatic lesion. Moreover, the patient has survived for more than 46 months with no evidence of loco regional recurrence or distant metastasis. Thus, we felt confident in supporting the diagnosis of PMMD.

Although the possibility of primary malignant melanoma in the small intestine does exist, the incidence is extremely low. In addition, melanoma by itself is a great mimicker of other neoplastic conditions [11]. It is very easy to diagnose for the cases with obvious melanin pigment, but sometimes, the specimen only with little pigment or without any visible pigment, which may create a major diagnostic challenge, must be differentiated from other common intestinal tumors like lymphoma, carcinoma of poor differentiation, neuroendocrine tumor, leiomyosarcoma, GIST (Gastrointestinal Stromal Tumor), et al. More sections as far as possible and careful observation can help to find valuable diagnositic clues. Furthermore, immunohistochemical staining and electron microscope detection may play an important role for differential diagnosis. In our patient, the initial frozen sections revealed the tumor cells were relatively uniform with markedly cytologic atypia and abundant mitotic figures, so the pathologists favored lymphoma or carcinoma of poor differentiation, which were the most common malignancies of the duodenum. But further immunohistochemical stains provided no evidence for above diagnoses. By more careful observation, we found the discohesive tumor cells had one or more large eosinophilic nucleoli, which were one of the main features of melanoma apart from visible melanin pigment, so malignant melanoma - a very rare malignancy in this site should be taken into consideration. Expression of some specific markers such as HMB45, Melan-A and S-100 protein is required to confirm our conjecture.

Optical treatment for malignant melanoma is an extensive and curative surgery, if possible, because other methods including adjuvant radiotherapy, chemotherapy and immunotherapy cannot offer definite treatment outcome. The time of diagnosis and the presence of metastases are supposed to represent major determinants of prognosis [10,12]. The median survival after curative resection of primary malignant melanoma of the small intestine is 49 months [7]; the longest reported survival is 21 years [12]. To the best of our knowledge, our patient refused chemotherapy and just used Chinese traditional medicine intermittently. Now he has achieved disease-free survival of more than 46 months without any evidence of recurrence. The certain mechanism of Chinese traditional medicine is still unclear and always be regarded as complicated, sometimes even mysterious. Nowadays, one of generally accepted opinions is that it can enhance patients’ immunity efficiently. But melanoma is destined to behave aggressively, and widespread metastases may present as a late complication. Furthermore, the Ki-67 labeling index in this case was greater than 60% and the patient had one mesentery lymph node involved. This highlights the need for close and long-term follow-up.

## Conclusion

In conclusion, PMMD is an extremely rare neoplastic lesion of the alimentary tract. There are no specific symptoms and radiologic imaging. Especially, the cases with little melanin pigment or without visible melanin pigment are very misleading. Definite diagnosis depends on not only pathomorphology and immunohistochemical stains, but also detailed history and thorough investigation, the latter are more important to exclude the preexistence or coexistence of a primary lesion elsewhere.

## Consent

Written informed consent was obtained from the patient for publication of this Case Report and any accompanying images. A copy of the written consent is available for review by the Editor-in-Chief of this journal.

## Abbreviations

PMMD: Primary malignant melanoma of the duodenum; GI: Gastrointestinal.

## Competing interests

The authors declare that they have no competing interests.

## Authors’ contributions

LH conceived and designed the case report and drafted the manuscript; ZZ designed the case report and contributed to the revisions and editing of the manuscript. FQ participated in the histopathological evaluation and gave the final diagnosis; WZ participated in the histopathological evaluation and supplied the literature review; XH provided the relevant radiological details; LX and ZW participated in immunohistochemical analysis. All authors have read and approved the final manuscript.
